# Chronic primary musculoskeletal pain: a new concept of nonstructural regional pain

**DOI:** 10.1097/PR9.0000000000001024

**Published:** 2022-08-09

**Authors:** Mary-Ann Fitzcharles, Steven P. Cohen, Daniel J. Clauw, Geoffrey Littlejohn, Chie Usui, Winfried Häuser

**Affiliations:** a Department of Rheumatology, McGill University, Montreal, QC, Canada; b Alan Edwards Pain Management Unit, McGill University, Montreal, QC, Canada; c Department of Anesthesiology and Critical Care Medicine, Neurology, Physical Medicine and Rehabilitation and Psychiatry and Behavioral Sciences, Johns Hopkins School of Medicine, Baltimore, MD, USA; d Uniformed Services University of the Health Sciences, Bethesda, MD, USA; e Departments of Anesthesiology, Medicine (Rheumatology), and Psychiatry, Chronic Pain and Fatigue Research Center, the University of Michigan Medical School, Ann Arbor, MI, USA; f Departments of Rheumatology and Medicine, Monash Health and Monash University, Clayton, Australia; g Department of Psychiatry, Juntendo University School of Medicine, Tokyo, Japan; h Department Internal Medicine I, Klinikum Saarbrücken, Saarbrücken, Germany; i Department of Psychosomatic Medicine and Psychotherapy, Technische Universität München, München, Germany

**Keywords:** Chronic, Pain, Musculoskeletal, Primary

## Abstract

The concept that a regional musculoskeletal pain may occur in the absence of identifiable tissue abnormality may be puzzling. Previously these regional complaints were generally categorized as myofascial pain syndromes, or prior to the formalization of the nociplastic pain concept, as musculoskeletal pain with a neuropathic component, and treatments were anatomically focussed. Chronic primary musculoskeletal pain is now identified under the chronic primary pain stem category with the mechanistic descriptor of nociplastic pain. It is possible that many patients previously diagnosed with myofascial pain do in fact suffer from chronic primary musculoskeletal pain, requiring a paradigm shift in management towards more centrally directed treatment strategies. Many questions remain, including validation of the proposed examination techniques, prevalence, ideal treatment, and uptake and acceptance by the healthcare community. This new classification should be welcomed as an explanation for regional pain conditions that previously responded poorly to physically focussed treatments.

**Associated commentaries:** Cohen ML. Clarifying “chronic primary musculoskeletal pain”? The waters remain murky. PAIN Reports 2022;7:e1021.

Treede R-D. Chronic musculoskeletal pain: traps and pitfalls in classification and management of a major global disease burden. PAIN Reports 2022;7:e1023.

Chronic pain conditions, especially those not supported by objective tissue damage, are a challenge to understand, diagnose, and treat. The recently published *International Statistical Classification of Diseases* and Related Health Problems (*ICD*) codes, *ICD-11* identifies chronic pain as a stem code, with chronic primary pain a subcategory that can occur in one or more anatomical regions independently of identifiable biological or psychological contributors.^19^ Contained within the chronic primary pain stem category are chronic widespread pain and chronic primary musculoskeletal (MSK) pain. These primary pain conditions have no identifiable tissue abnormalities, with central sensitization proposed as a mechanistic etiology. Distinct from nociceptive or neuropathic pain, these pain conditions can be categorized as a mechanistic third pain descriptor termed nociplastic pain.^11^ Best studied in patients with fibromyalgia syndrome (FMS), central sensitization, with additional neurobiological or psychosocial mechanisms, likely explains chronic primary pain conditions.^6^

The concept of MSK pain, understood as pain arising in muscles, tendons, bones, and joints, with absent anatomical change may be baffling, warranting elucidation and investigation. In simplistic terms, chronic primary MSK pain is best understood as “regional fibromyalgia,” with pathoanatomical differences but clinical implications.^10^ Regional MSK complaints are commonly recognized as myofascial pain syndromes (MPS), or before formalization of the nociplastic pain concept, as MSK pain with a “neuropathic component.” This article aims to create dialogue regarding similarities and differences between chronic primary MSK pain and MPS.

## 1. Chronic primary musculoskeletal pain

Chronic pain, widely recognized as teleologically distinct from acute pain, is a symptom portending tissue damage. Most extensively studied in the nonregional prototype fibromyalgia, chronic pain without tissue damage frequently affects physical, emotional, and social functioning.^1,17^ Within the *ICD-11* framework, chronic primary MSK pain falls under the umbrella of “chronic primary pain,” persisting for more than 3 months, with significant emotional distress or functional disability as viewed within a biopsychosocial framework, and cannot be accounted for by another condition.”^14^ The pain associates of sleep disturbances, fatigue, disturbed memory and mood, and hypersensitivity phenomena are heterogenous and unpredictable. The final phenotypic expression is modulated by psychosocial factors. Compared with other forms of pain, the negative impact on self-esteem, interpersonal relationships, and financial status is often more pronounced, with further stigmatization because of its “invisible” nature.^2,9^

Regional pain syndromes have been recognized by astute clinicians in the past. In 2007, Littlejohn wrote “Regional pain syndrome is characterized by regional pain and tenderness, which has a non-neuroanatomic distribution, and shares clinical features with both fibromyalgia syndrome and complex regional pain syndrome”, similarities now reflected in the *ICD-11*, and with psychosocial rather than ergonomic factors contributing to symptoms.^12^ This concept received scant attention, with treatments mostly focused on perceived anatomical changes less relevant to the underlying physiological process (biomedical vs biopsychosocial approach). Chronic primary MSK pain now introduces the concept that not all regional pain conditions are due solely to tissue abnormalities, but that some aspects can be mechanistically explained as sensitization of the nervous system. The notion that pain previously attributed exclusively to structural pathology may predominantly be due to disordered pain processing is supported by research demonstrating that a substantial proportion of patients with orthopedic conditions actually possess mixed pain phenotypes.^8^

The new classification of chronic primary MSK pain provides an opportunity to categorize, diagnose, and treat musculoskeletal pain conditions previously referred to as “nonspecific.”^14^ In a recent review, Kosek et al. proposed 4 clinical criteria to identify nociplastic pain affecting the musculoskeletal system (chronic primary MSK pain), although some have objected to these criteria and the authors acknowledge that they still required validation.^10^ A possible diagnosis requires the presence of 2 criteria (pain and an examination finding) while a probable diagnosis requires that all 4 (pain, an examination finding, pain sensitivity to external stimuli, and central symptoms) be present. These criteria do not represent a new diagnostic category but rather a mechanistic explanation for MSK pain conditions that to date have defied clear understanding.

A **possible** diagnosis of chronic primary MSK pain requires the following: (1) Pain is regional (not discrete), present for at least 3 months and cannot entirely be explained by associated neuropathic or nociceptive pain **and** (2) clinical examination demonstrating evoked pain hypersensitivity. A **probable** diagnosis requires “1” and “2” as well as: (3) history of pain sensitivity to touch, pressure, movement, or temperature **and** (4) presence of hypersensitivity to sound or light or odours, sleep disturbances, fatigue, or cognitive problems.

## 2. Is chronic primary musculoskeletal pain different from myofascial pain syndrome?

In assigning a diagnosis of chronic primary MSK pain, attention should be paid to another regional pain syndrome, MPS.^18^ Early understanding of MPS alluded to myofascial abnormalities, although subsequent study has not identified consistent evidence of local tissue pathology.^3,16^ This syndrome is further fraught with controversy because there is currently no universal consensus on the etiology, pathology, diagnostic criteria, or ideal treatment. Proposed etiologies cover a wide range of mechanisms including regional soft tissue inflammation, hypercontracted muscle, sustained low-level muscular exertion, and peripheral and central sensitization. Diagnosis is based on symptom characteristics and examination manoeuvres. The overlap between these 2 diagnoses should prompt consideration that they may not be distinct but rather share common underlying mechanisms.

Similarities between these 2 conditions include non-neuroanatomical pain distribution and presence of central and constitutional symptoms such as fatigue, vegetative symptoms (eg, inattention, dyspepsia), mood and sleep disturbances, sensitization, and modulation by psychosocial factors.^3,4^ For MPS, physical abnormalities identified on muscle palpation include trigger points, tenderness, and a localized twitch response, all of which are subjective, operator dependent, and neither standardized nor reliable.^16^ Demonstration of findings is often used to justify treatments such as trigger point injections. Moreover, the examination skills required to identify chronic primary MSK pain are outside the realm of the average practicing clinician.

## 3. Challenges and value of diagnosing chronic primary musculoskeletal pain

This proposed new concept outlining that many MSK conditions previously attributed exclusively to musculoskeletal tissue pathology may be at least partially due to abnormalities in pain processing and will undoubtedly raise many questions and stimulate debate but is critical for establishing treatment paradigms. Some questions include the following:

(1) Is chronic primary MSK pain initiated peripherally? If so, when does the pain mechanism cross the boundary from nociceptive to nociplastic pain?
(2) Are there early predictors that influence transition to chronic pain sensitization?
(3) Are some locations and people more susceptible to pain sensitization and chronicity and can they be identified?
(4) How easily will physicians be able to distinguish primary from secondary MSK pain? Table 1^5^
(5) How willingly will patients and health care professionals accept treatments with a psychosocial rather than peripherally directed focus (although the ideal treatment may be a hybrid model)?

**Table 1:** Proposed distinguishing characteristics of primary and secondary musculoskeletal pain.^5^

Clinical characteristic	Secondary musculoskeletal pain* (predominantly nociceptive)	Primary musculoskeletal pain (predominantly nociplastic)
Etiology	Potential or actual tissue damage	Dysfunctional processing of pain and other sensory stimuli without tissue injury
Descriptors	Throbbing, aching, pressure-like	Sharp, shooting, lancinating, burning, aching
Sensory deficits	Infrequent	Common, in nonanatomical distribution
Motor deficits	May have pain-induced weakness	Generalized fatigue common; weakness may be related to deconditioning
Diagnostic tests	Imaging may show structural changes, but specificity is low. Laboratory tests also lack specificity.	Imaging and laboratory tests generally within normal limits; can rule out other sources of pain (eg, inflammatory arthritis)
Hypersensitivity	Uncommon except for hypersensitivity in the immediate area	Common, sometimes diffuse
Pain pattern	Distal radiation uncommon, referred pain if proximal structure involved	More diffuse and variable, not following anatomical referral pattern
Precipitating or relieving factors	Exacerbations less common and often associated with activity	Common, often related to psychosocial stress
Autonomic signs	Uncommon	Signs of autonomic dysfunction may be present
Quality of life changes	Quality of life decrements often less than for neuropathic pain	Quality of life decrements similar to or greater than for neuropathic pain
Concomitant conditions	Generally less psychopathology	Higher rates of psychopathology, cognitive impairment, and other comorbid pain conditions than for nociceptive or neuropathic pain

Categories subject to significant heterogeneity and variability.

* Includes conditions such as myofascial pain syndromes involving trigger points or abnormal myoelectric activity, inflammatory and noninflammatory arthritis, and soft tissue rheumatic complaints (eg, tendonitis).

There will undoubtedly be resistance to the concept of central pain sensitization as an explanation for some regional pain conditions by health care professionals who perform procedures, as well as financial ramifications if payers refuse to authorize them.

Many patients with persistent and unremitting pain will likely eventually be evaluated in pain management centers. Our concern is for persistent misdiagnosis of MPS, with treatments focused entirely on a localized anatomical process. Nociplastic pain requires multimodal management that includes centrally acting medications, self-management strategies, increased physical activity, and psychological therapies.^7,13^ By contrast, MPS and chronic secondary musculoskeletal pain management should be more peripherally focused with physical therapies, peripherally acting medications, and invasive treatments such as injections.^16^ We anticipate some resistance towards a biopsychosocial approach that is less operator-dependent by health care professionals practicing predominantly interventional medicine. The lack of consistency surrounding MPS has hindered clinical understanding, with current literature more focused on interventional approaches rather than understanding of pathogenesis.^15^

Attention to patients' perspectives is vital. Patients may have difficulty understanding that a pain condition, seemingly anatomically focused, has an important central component, especially when receiving solely peripherally focused treatments. Although patients intuitively seek a structural explanation for pain, it is hoped that with education, many will be relieved to have a plausible explanation for symptoms, understand why injections did not work, and be willing to accept more centrally directed nonpharmacological interventions.

## 4. Conclusions

The evolving concept of nociplastic pain is still in the process of gaining widespread acceptance and requires further validation and buy-in from nonpain specialists. Despite distinct phenotypic differences, the classification of chronic primary MSK pain, with a predominant nociplastic mechanism, may be easiest understood as “regional fibromyalgia,” a novel concept that will require acceptance by mainstream medicine. Purists may take issue with the term fibromyalgia because current criteria for FMS require widespread pain, which is not the case for regional nociplastic pain. We suggest retaining the term “fibromyalgia” to allow for better understanding and acceptance by the health care community. The value of diagnosing chronic primary MSK pain is to reassure patients, reduce unnecessary investigations, and shift the focus towards centrally rather than peripherally directed treatments. As a community, we must unite in support for the concept of chronic primary MSK pain and press forward with investigations to elucidate prevalence, identify mechanisms, and discover effective treatments.

## Disclosures

S.P. Cohen is supported by a grant from the U.S. Dept. of Defense, Uniformed Services University, Department of Physical Medicine & Rehabilitation, Musculoskeletal Injury Rehabilitation Research for Operational Readiness (MIRROR) (HU00011920011). S.P. Cohen has consulted for Avanos, Persica, SPR, and Scilex/Sorrento. D.J. Clauw has performed consulting for Pfizer, Tonix, Samumed, Lilly, and Aptinyx and has received research funding from Aptinyx. M.-A. Fitzcharles and SP Cohen contributed equally to this article. All authors participated in the writing of this manuscript. The remaining authors have no conflicts of interest to declare.
